# Protocol for rapid manipulation of mitochondrial morphology in living cells using inducible counter mitochondrial morphology (iCMM)

**DOI:** 10.1016/j.xpro.2021.100721

**Published:** 2021-08-05

**Authors:** Takafumi Miyamoto, Song-Iee Han, Hitoshi Shimano

**Affiliations:** 1 Department of Internal Medicine (Endocrinology and Metabolism), Faculty of Medicine, University of Tsukuba, Tsukuba, Ibaraki 305-8575, Japan; 2 Transborder Medical Research Center, University of Tsukuba, Tsukuba, Ibaraki 305-8577, Japan

**Keywords:** Cell Biology, Molecular/Chemical Probes, Protein Biochemistry, Biotechnology and bioengineering

## Abstract

Disruption of mitochondrial morphology occurs during various diseases, but the biological significance is not entirely clear. Here, we describe a detailed step-by-step protocol for a chemically inducible dimerization system-based synthetic protein device, termed inducible counter mitochondrial morphology. This system allows artificial manipulation of mitochondrial morphology on a timescale of minutes in living mammalian cells. We also describe an AI-assisted imaging processing approach.

For complete details on the use and execution of this protocol, please refer to Miyamoto et al., 2021.

## Highlights

• Protocol for rapid manipulation of mitochondrial morphology by iCMM
• iCMM is a genetically encoded chemically inducible dimerization system
• AI-assisted image processing for mitochondrial morphology

## Graphical abstract

**Figure:**
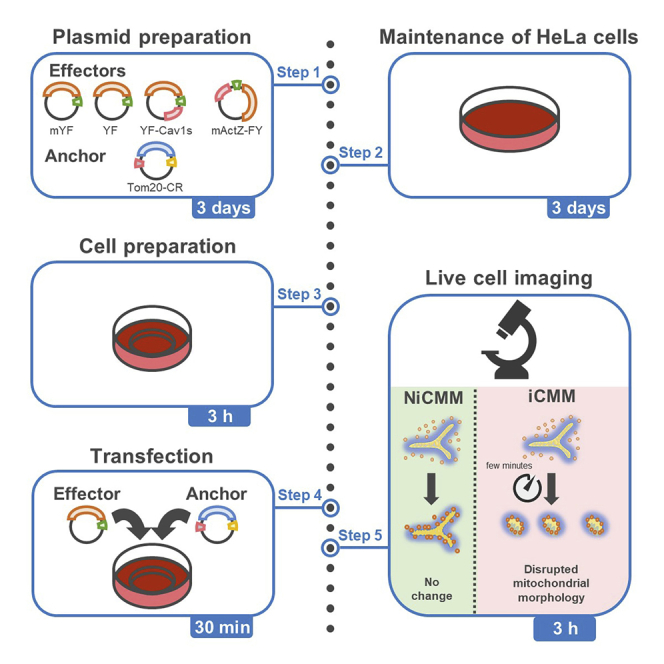

## Before you begin

In this paper, we describe a step-by-step protocol for manipulating mitochondrial morphology in living cells at any given time point within a few minutes using iCMM, a Boolean YES logic gate-based synthetic protein device based on a Chemically Inducible Dimerization (CID) system. When this protocol is followed, mitochondrial morphological changes are observed within 1–5 min of actuating the iCMM. It employs the CID method with rapamycin as a chemical dimerizer; however, the fundamental experimental procedure is the same when other CID methods are used.

**Figure 1:**
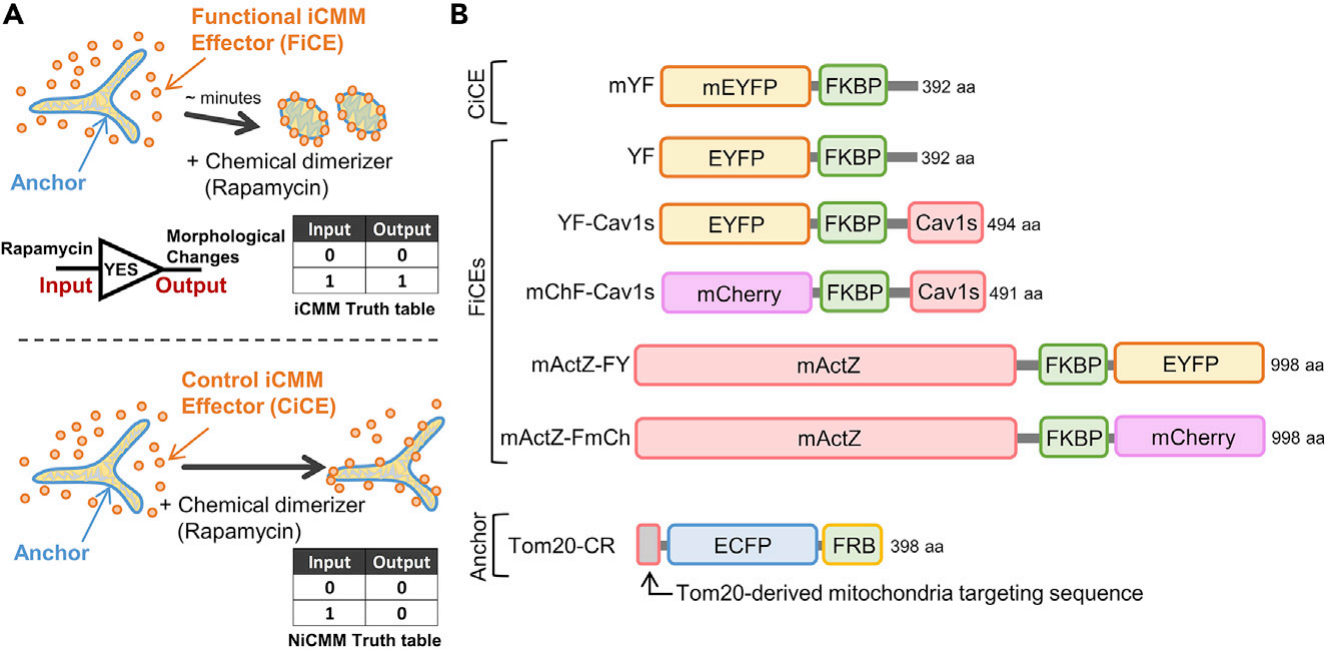
Schematic diagram of iCMM and NiCMM Reprinted with permission from Miyamoto et al., 2021. (A) Schematic diagram of the iCMM and NiCMM system. (B) Structural features of functional iCMM effectors (FiCEs), control iCMM effector (CiCE), and anchor.

The iCMM consists of a functional iCMM effector (FiCE), which can induce changes in mitochondrial morphology, and a mitochondrial outer membrane-specific anchor protein that tethers the effector to the mitochondria in the presence of a chemical dimerizer (Figure 1A). In contrast, an effector that does not cause mitochondrial morphological changes is termed a control iCMM effector (CiCE), and a synthetic protein device consisting of a CiCE and a mitochondria-specific anchor is referred to as a negative iCMM (NiCMM; Figure 1A).

To date, five FiCEs (YF, YF-Cav1s, mChF-Cav1s, mActZ-FY, and mActZ-FmCh), one CiCE (mYF), and one anchor (Tom20-CR) (Miyamoto et al., 2021) have been developed (Figure 1B). The effector (FiCE/CiCE) and anchor contain interaction domains (in this protocol, FKBP and FRB; Figure 1B) required to induce dimer formation through chemical dimerizers (Derose et al., 2013). It is possible to create various effectors and anchors with different characteristics by reorganizing protein structures according to the intended purpose.

The main characteristic of iCMM is that it can induce morphological changes in mitochondria on a minute timescale at any time point. Thus, changes that occur in the cell immediately after induction of mitochondrial morphological changes can be observed.

### Plasmid preparation for transfection

**Timing: 3 days**

1. Expression plasmids encoding effectors and anchor should be prepared in advance. These plasmids are available from Addgene (Watertown, MA, USA; https://www.addgene.org/).
2. Day 1: transform plasmids into competent cells as follows:
  a. Thaw ECOS Competent *E. coli* DH5α cells (NIPPON GENE, Tokyo, Japan) on ice.
  b. Add 10 ng of plasmid to 20 μL competent cells in a 1.5-mL microcentrifuge tube and mix gently.
  c. Incubate the mixture on ice for 20 min.
  d. Heat shock at 42°C for 45 s.
  e. Put the mixture on ice for 3 min.
  f. Add 50 μL LB liquid medium without antibiotics to the mixture.
  g. Incubate the mixture at 37°C for 60 min.
  h. Warm LB agar plates containing 50 μg/mL kanamycin at 37°C for 60 min.
  i. Spread all of the mixture onto the LB agar plates containing 50 μg/mL kanamycin.
  j. Incubate the plates at 37°C overnight (16–18 h).

***Alternatives:*** Other competent cells, such as the *E. coli* DH5α Competent Cells (TaKaRa, 9057), can be used instead of ECOS Competent *E. coli* DH5α cells.

3. Day 2: select a single clone and culture it in 100 mL LB medium containing 50 μg/mL kanamycin in a 200-mL flask.
4. Shake at 37°C and 160 rpm overnight (16–18 h).
5. Day 3: transfer the bacterial culture to two 50-mL conical tubes and centrifuge at 4,000 × *g* and 4°C for 10 min.
6. Remove the supernatant.

**Pause point:** Bacterial pellets can be stored at −80°C until purification.

7. Purify the plasmid for transfection using the PureLink™ HiPure Plasmid Midiprep Kit (Thermo Fisher Scientific, Waltham, MA, USA) according to the manufacturer’s instructions (https://www.thermofisher.com/document-connect/document-connect.html?url=https%3A%2F%2Fassets.thermofisher.com%2FTFS-Assets%2FLSG%2Fmanuals%2Fpurelink_hipure_plasmid_dna_purification_man.pdf&title=VXNlciBHdWlkZTogUHVyZUxpbmsgSGlQdXJlIFBsYXNtaWQgRE5BIFB1cmlmaWNhdGlvbiBLaXRz).

***Note:*** Plasmid DNA at a A260/A280 ratio < 1.8 should be used at a concentration of 1 mg/mL.

***Alternatives:*** Other plasmid purification kits, such as the QIAGEN Plasmid Midi Kit (QIAGEN, 12143) or NucleoBond™ Xtra Midi (TaKaRa, 740410.10), may be used to purify plasmids according to the manufacturer’s instructions (https://www.qiagen.com/us/products/discovery-and-translational-research/dna-rna-purification/dna-purification/plasmid-dna/qiagen-plasmid-kits/?catno=12143 or https://www.mn-net.com/media/pdf/ed/82/0f/Instruction-NucleoBond-Xtra.pdf).

### Maintenance of HeLa cells

**Timing: 3 days**

Human cervical adenocarcinoma HeLa cells were purchased from the American Type Culture Collection (Manassas, VA, USA) and were cultured in Dulbecco’s modified Eagle’s medium (DMEM) supplemented with 10% fetal bovine serum (FBS) and 1% ZellShield (Minerva Biolabs, Berlin, Germany) at 37°C in 5% CO_2_.

***Alternatives:*** ZellShield (Minerva Biolabs) can be substituted for penicillin-streptomycin (final conc. 100 units penicillin and 0.1 mg streptomycin/mL).

***Alternatives:*** iCMM can be used with cells other than HeLa cells. We confirmed that iCMM can induce mitochondrial morphological changes in Hep 3 B human hepatoma and U-2 OS human osteogenic sarcoma cells (Miyamoto et al., 2021).

8. Day 1: thaw cells stored in a −80°C freezer or in liquid nitrogen were transfered to a 15-mL conical tube containing 10 mL prewarmed culture medium.

***Note:*** CELLBANKER (Nippon Zenyaku Kogyo, Tokyo, Japan), a ready-to-use cell cryopreservation medium, may be used for cell stocks.

9. Centrifuge the tube with swing buckets at 161 × *g* and at room temperature (15°C–25°C) for 2 min.

***Note:*** Sorvall ST 8 (Thermo Fisher Scientific) may be used for centrifugation.

10. Remove the supernatant and resuspended precipitated cells in 10 mL prewarmed culture medium.
11. Plate the cell suspension in a 10-cm cell culture dish.

***Note:*** After plating cells in the culture dish, use a microscope to ensure that the cells are not dead. Furthermore, 24 h after plating verify that confluency is < 50%; if not, then proceed to Step 13 (Day 3).

12. Incubate cells at 37°C and 5% CO_2_ for 48 h.
13. Day 3: remove the culture medium.
14. Add 4 mL prewarmed phosphate-buffered saline (PBS) at the periphery of the dish and slowly tilt to gently wash the cells.
15. Remove PBS.
16. Add 1 mL 0.05% trypsin-EDTA solution to the dish.
17. Incubate the cells at 37°C for 4 min in a 5% CO_2_ incubator.
18. Gently tap the dish to completely detach the cells.
19. Add 7 mL prewarmed culture medium and suspend the cells by gentle pipetting.
20. Transfer the cells to a sterilized 15-mL conical tube.
21. Centrifuge the tube with swing buckets at 161 × *g* and at room temperature (15°C–25°C) for 2 min.
22. Add 7 mL prewarmed culture medium and suspend the cells by gentle pipetting.
23. To count cells, mix 40 μL of the cell suspension with 40 μL trypan blue in a 1.5-mL microcentrifuge tube. Pipette up and down gently several times to mix.
24. Use an automated cell counter with 10 μL of the mixture.

***Alternatives:*** A hemocytometer may be used for cell counts.

***Note:*** Verify, at least qualitatively, that cell viability, shape, and size are not atypical. If the cell counter used can measure quantitative information about viability, size, among others, then it is recommended to record such numerical information.

25. Plate 1 × 10^6^ cells in a 10-cm dish and culture at 37°C under 5% CO_2_.

***Note:*** In this case (1 × 10^6^ cells in a 10-cm dish), seeded HeLa cells need to be passaged after 48 h. To passage cells at 72-h intervals, plate 0.5 × 10^6^ cells in a 10-cm dish. The number of cells to be plated should be changed according to the cells’ growth rate. Confluency of 80% is recommended as a standard for cell passaging. To prevent damage to the cells, we do not recommend passaging at 24-h intervals.

**CRITICAL:** Before starting the experiment, infection of the cells with Mycoplasma should be excluded using PCR and Hoechst staining. To examine whether cells are infected with Mycoplasma by PCR, we recommend using ready-to-use kits such as e-MycoTM Mycoplasma PCR Detection Kit (iNtRON Biotechnology, Cat#25235), e-MycoTM VALiD Mycoplasma PCR Detection Kit (iNtRON Biotechnology, Cat#25239), TaKaRa PCR Mycoplasma Detection Set (Takara, Cat#6601), or LookOut® Mycoplasma PCR Detection Kit (Sigma-Aldrich, Cat#MP0035-1KT). For Hoechst staining-based Mycoplasma contamination test, please refer to the technical article at the following site: https://www.sigmaaldrich.com/JP/en/technical-documents/technical-article/microbiological-testing/mycoplasma-testing/testing-for-mycoplasma.

## Key resources table

**Table:** 

REAGENT or RESOURCE	SOURCE	IDENTIFIER
**Bacterial strains**		

ECOS Competent *E. coli* DH5α	NIPPON GENE	Cat# 312-07031

**Chemicals**		

Dulbecco’s Modified Eagle’s Medium (DMEM)	Thermo Fisher Scientific	Cat# 11965118
Phenol red-free DMEM	Thermo Fisher Scientific	Cat# 31053028
Fetal Bovine Serum (FBS)	Thermo Fisher Scientific	Cat# 10270-106
l-glutamine	Thermo Fisher Scientific	Cat# 25030081
Phosphate-Buffered Saline (PBS)	Thermo Fisher Scientific	Cat# 10010023
ZellShield	Minerva Biolabs GmbH	Cat# 13-0050
Penicillin-Streptomycin	Sigma-Aldrich	Cat# P4333
0.05% Trypsin-EDTA Solution	Thermo Fisher Scientific	Cat# 25300-054
Opti-MEM	Thermo Fisher Scientific	Cat# 11058-021
FuGENE HD	Promega	Cat# E2311
MitoTracker Red CMXRos	Thermo Fisher Scientific	Cat# M7513
Rapamycin	Calbiochem	Cat# 553211-1MGCN
Trypan Blue Stain	Thermo Fisher Scientific	Cat# 15250-061
Hoechst 33342, Trihydrochloride, Trihydrate	Thermo Fisher Scientific	Cat# H1399
CELLBANKER	Nippon Zenyaku Kogyo	Cat# CB023
STAR Agar L-grade	Rikaken	Cat# RSU-AC01-500G
Tryptone	Nacalai Tesque	Cat# 35640-95
Extract Yeast Dried	Nacalai Tesque	Cat# 15838-45
NaCl	Nacalai Tesque	Cat# 31320-05
Kanamycin Sulfate	FUJIFLIM	Cat# 117-00341

**Critical commercial assays**		

PureLink™ HiPure Plasmid Midiprep Kit	Thermo Fisher Scientific	Cat# K210005
e-Myco^TM^ Mycoplasma PCR Detection Kit (ver.2.0)	iNtRON Biotechnology	Cat# 25235

**Experimental models: Cell lines**		

HeLa	ATCC	CCL-2

**Recombinant DNA**		

mYF	Miyamoto et al., 2021	Addgene Plasmid #171460
YF	Miyamoto et al., 2021	Addgene Plasmid #171459
YF-Cav1s	Miyamoto et al., 2021	Addgene Plasmid #171462
mActZ-YF	Miyamoto et al., 2021	Addgene Plasmid #171463
Tom20-CR	Miyamoto et al., 2021	Addgene Plasmid #171461

**Software and algorithms**		

NIS-Elements AR 5.30	Nikon	https://www.nsl.nikon.com/

**Other**		

3.5-cm poly-lysine-coated glass bottom dish	Matsunami	Cat# D1131H
10-cm cell culture dish	Corning	Cat# 430167
15-mL Conical Tube	Thermo Fisher Scientific	Cat# 239650
50-mL Conical Tube	Thermo Fisher Scientific	Cat# 339652
Sterilized petri dish	WiSM	Cat# WMS-500
1.5-mL microcentrifuge tube	BIO-BIK	Cat# CF-0150
5-mL polystyrene round-bottom tube	?	?
Toothpicks	N/A	N/A
Water bath (thermo minder SD mini)	TAITEC	Cat# 0034942-000
CO_2_ incubator (37°C, 5% CO_2_)	Panasonic	Cat# MCO-170AICUV-PJ
Luna^TM^ automated cell counter	Shinkouseiki.Co.,Ltd.	Cat# L10001
NanoDrop^TM^ (ND-1000 spectrophotometer)	Thermo Fisher Scientific	Cat# ND-1000
Eclipse Ti2-E microscope and Intensilight mercury-fiber illuminator	Nikon	https://www.microscope.healthcare.nikon.com/products/inverted-microscopes/eclipse-ti2-series/eclipse-ti2-e
CFP-A-Basic-NTE filter	Semrock	http://www.opto-line.co.jp/sem/detail_sets.html?pn=CFP-A-Basic
YFP-A-Basic-NTE filter	Semrock	http://www.opto-line.co.jp/sem/detail_sets.html?pn=YFP-A-Basic
mCherry-B-NTE-ZERO filter	Semrock	http://www.opto-line.co.jp/sem/detail_sets.html?pn=mCherry-C
Plan Apochromat Lambda Series, 40× objective	Nikon	https://www.microscope.healthcare.nikon.com/products/optics/cfi-plan-apochromat-lambda-series
Zyla 4.2 PLUS sCMOS camera	Oxford Instruments	https://andor.oxinst.jp/products/scmos-camera-series/zyla-4-2-scmos
STX stage top incubator	Tokai Hit	https://www.tokaihit.com/about-stage-top-incubator/

## Materials and equipment

### Plasmid preparation for transfection

**Table:** LB agar plates containing 50 μg/mL kanamycin

Reagent	Final concentration	Amount
Tryptone	1%	1 g
Dried yeast extract	0.5%	0.5 g
NaCl	1%	1 g
STAR Agar L-grade	1.5%	1.5 g
MilliQ water	n/a	up to 100 mL
50 mg/mL kanamycin	50 μg/mL	100 μL
**Total**	**n/a**	**100 mL**

***Note:*** Please refer to the addgene website (https://www.addgene.org/protocols/pouring-lb-agar-plates/) for detailed preparation instructions.

***Note:*** The LB agar plates can be stored at 4°C away from light until use. They should be used within a month.

**Table:** LB liquid medium containing 50 μg/mL kanamycin

Reagent	Final concentration	Amount
Tryptone	1%	1 g
Dried yeast extract	0.5%	0.5 g
NaCl	1%	1 g
MilliQ water	n/a	up to 100 mL
50 mg/mL kanamycin	50 μg/mL	100 μL
**Total**	**n/a**	**100 mL**

***Note:*** Please refer to the addgene website (https://www.addgene.org/protocols/inoculate-bacterial-culture/) for detailed preparation instructions.

***Note:*** The medium can be stored at room temperature (15°C–25°C) without antibiotics until use. Thus, the LB liquid medium can be prepared a day before its intended use.

### Maintenance of HeLa cells

Culture medium (DMEM supplemented with 10% FBS and 1% ZellShield)

**Table:** 

Reagent	Final concentration	Amount
DMEM	n/a	445 mL
FBS	10%	50 mL
ZellShield	1%	5 mL
**Total**	**n/a**	**500 mL**

***Note:*** The culture medium can be stored at 4°C away from light until use.

### Live cell imaging

- To prepare a stock solution of rapamycin, dissolve rapamycin in dimethyl sulfoxide to a final concentration of 100 μM. Stock solutions may be stored at −30°C until use.
- For live cell imaging, prepare phenol red-free DMEM supplemented with 10% FBS, 4 mM L-glutamine, and 1% penicillin and streptomycin (referred to as imaging medium).

**Table:** Imaging medium

Reagents	Final concentration	Amount
Phenol red-free DMEM	n/a	435 mL
FBS	10%	50 mL
l-glutamine (200 mM)	4 mM	10 mL
Penicillin-streptomycin	100 units penicillin and 0.1 mg streptomycin/mL	5 mL
**Total**	**n/a**	**500 mL**

***Note:*** The imaging medium can be stored at 4°C away from light until use.

## Step-by-step method details

### Seeding and culturing of the cells (day 1)

**Timing: 3 h**

Before starting this protocol, cells should be cultured for at least one week after thawing; additionally, the confluency of the cells should not exceed 80%.

1. Add 4 mL prewarmed PBS at the periphery of the dish and slowly tilt to gently wash the cells.
2. Remove PBS.
3. Add 1 mL 0.05% trypsin-EDTA solution to the dish.
4. Incubate the cells at 37°C for 4 min in a 5% CO_2_ incubator.
5. Gently tap the dish to completely detach the cells.
6. Add 5 mL prewarmed fresh culture medium and suspend the cells by gentle pipetting.
7. Transfer the cells to a sterilized 15-mL conical tube.
8. Centrifuge the tube with swing buckets at 161 × *g* and at room temperature (15°C–25°C) for 2 min.
9. Add 7 mL prewarmed culture medium and suspend the cells by gentle pipetting.
10. To count cells, mix 40 μL of the cell suspension with 40 μL trypan blue in a 1.5-mL microcentrifuge tube. Gently pipette up and down several times to mix.
11. Use an automated cell counter with 10 μL of the mixture.

***Alternatives:*** A hemocytometer may be used for cell counts.

***Note:*** Verify, at least qualitatively, that cell viability, shape, and size are not atypical. If the cell counter used can measure quantitative information about viability and size, among others, it is recommended to record such numerical information.

12. Plate 2.4 × 10^5^ cells in a 3.5-cm poly-lysine-coated glass bottom dish and culture at 37°C in 5% CO_2_ (2 mL/dish).

***Alternatives:*** A 3.5-cm poly-lysine-coated glass-bottom dish can be substituted for either glass cover slips coated with poly-D-lysine or a 35-mm imaging dish with a polymer coverslip bottom (e.g., iBidi, Fitchburg, WI, USA; cat# 81156). Notably, as changes in cell adhesion conditions may alter cellular responses to various factors, it is recommended that cell adhesion conditions be kept consistent.

13. After plating, culture cells at 37°C and 5% CO_2_ in an incubator for 4 h (until cells attach well) and then transfect.

### Transfection (day 1)

**Timing: 30 min**

14. Transfect as follows:
  a. Add 0.5 μg of each plasmid-encoding effector and anchor (1:1 ratio; total 1 μg) to 100 μL Opti-MEM (Thermo Fisher Scientific) in a 5-mL polystyrene round-bottom tube.
  b. Add 3 μL FuGENE HD (Promega) and mix gently to avoid introducing bubbles.
  c. Incubate the transfection mixture at room temperature (15°C–25°C) for 10 min.
  d. Add 100 μL of the mixture to the cells that are plated on a 3.5-cm poly-lysine-coated glass-bottom dish and mix gently.

***Note:*** The amount and proportion of plasmids need to be optimized according to the used cell line, the transfection reagents, and the purpose of the experiment.

***Alternatives:*** Other transfection reagents such as Lipofectamine^TM^ 3000 Transfection Reagent (Thermo Fisher Scientific, L3000001) may be used to transfect cells according to the manufacturer’s instructions (https://www.thermofisher.com/document-connect/document-connect.html?url=https%3A%2F%2Fassets.thermofisher.com%2FTFS-Assets%2FLSG%2Fmanuals%2Flipofectamine3000_protocol.pdf&title=TGlwb2ZlY3RhbWluZSAzMDAwIFJlYWdlbnQgUHJvdG9jb2wgKEVuZ2xpc2gp).

15. After transfection, culture cells under 5% CO_2_ at 37°C for 20–32 h.

### Live cell imaging using epifluorescence microscopy (day 2)

**Timing: 2 h**

Cells were viewed using a 40× objective (Plan Apochromat Lambda Series, Nikon) mounted on an inverted Eclipse Ti2-E microscope (Nikon) and imaged using a Zyla 4.2 PLUS sCMOS camera (Oxford Instruments). Imaging data were processed using the NIS-Elements AR imaging software (Nikon).

16. Two hours before live cell imaging, replace culture medium containing the transfection mixture with 2 mL prewarmed fresh culture medium.

***Note:*** To reduce cytotoxicity of transfection reagents, the culture medium should be replaced at least 12–16 h after transfection.

17. Activate the STX stage top incubator system (Tokai Hit, Fujinomiya, Japan) to maintain a temperature of 37°C in 5% CO_2_ for 1 h before live cell imaging.
18. Add 1 μL 1 mM MitoTracker Red CMXRos (Thermo Fisher Scientific) into the glass-bottom dish (final concentration: 500 nM) 30 min before live cell imaging.

***Note:*** To stain the cells uniformly, 1 μL MitoTracker Red CMXRos may be aliquoted to a 1.5-mL microcentrifuge tube and suspended in 100 μL culture medium. The suspension may then be added to the dish followed by gentle agitation. Additionally, the concentration of MitoTracker Red CMXRos should be optimized for each cell line used according to the manufacturer’s protocol (https://www.thermofisher.com/document-connect/document-connect.html?url=https%3A%2F%2Fassets.thermofisher.com%2FTFS-Assets%2FLSG%2Fmanuals%2Fmp07510.pdf&title=VXNlciBHdWlkZTogTWl0b1RyYWNrZXIgTWl0b2Nob25kcmlvbi1TZWxlY3RpdmUgUHJvYmVz).

**Figure 2:**
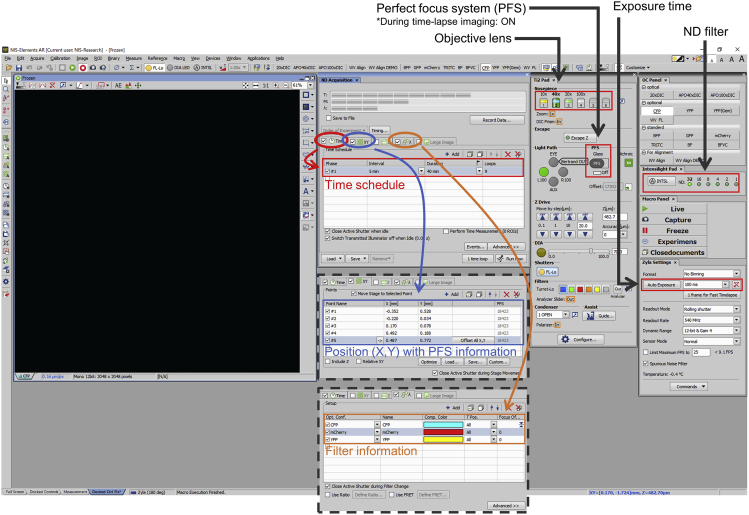
An example of acquisition setting in the NIS-Elements AR imaging software Details of acquisition settings in the NIS-Elements AR imaging software are shown.

***Note:*** Although we do not have strict criteria for determining the imaging positions, we believe it is important to select the imaging positions so that we can capture iCMM/NiCMM-expressing cells without bias as much as is possible.

***Note:*** Live cell imaging was performed under the same imaging conditions for each experiment. Representative imaging conditions are described below.

19. During the staining process, start the microscopy system according to the manufacturer’s instructions (https://www.microscope.healthcare.nikon.com/products/inverted-microscopes/eclipse-ti2-series/eclipse-ti2-e).
20. Thirty minutes after adding MitoTracker Red CMXRos, remove the culture medium and wash twice using 1 mL prewarmed imaging medium.
21. Add 2 mL prewarmed imaging medium.
22. Find and focus cells expressing NiCMM/iCMM.
23. Select five positions for live cell imaging.
24. Set the imaging parameter of the NIS-Elements AR imaging software (Nikon) (Figure 2)

#### General information

Camera Name: Andor Zyla VSC-07685

Numerical Aperture: 0.95

No Z-stack imaging

#### mCherry (for mitochondria imaging)

Modality: Widefield Fluorescence

Filter: mCherry-B-NTE-ZERO filter (excitation: 542–582 nm, emission: 604–679 nm)

Binning: 1×1

Exposure: 100 ms

Perfect focus (PFS), state: On

Zoom: 1.00×

Intensilight, Illuminator(Illuminator (EPI)) NDFilter: 3.1

#### ECFP (for anchor imaging)

Modality: Widefield Fluorescence

Filter: CFP-A-Basic-NTE filter (excitation: 425.5–442.5 nm, emission: 459–499 nm)

Binning: 1×1

Exposure: 100 ms

PFS, state: On

Zoom: 1.00×

Intensilight, Illuminator (Illuminator (EPI)) NDFilter: 3.1

#### EYFP (for effector imaging)

Modality: Widefield Fluorescence

Filter: YFP-A-Basic-NTE filter (excitation: 489–505 nm, emission: 524–546 nm)

Binning: 1×1

Exposure: 100 ms

PFS, state: On

Zoom: 1.00×

Intensilight, Illuminator (Illuminator (EPI)) NDFilter: 3.1

25. Start time-lapse imaging.

***Note:*** The interval between image acquisition is changed depending on the purpose of the experiment. Two representative imaging schedules are shown below.

[Schedule 1] Interval: 2 min, Duration: 40 min, Adding chemical dimerizer: frame 6

[Schedule 2] Interval: 5 min, Duration: 60 min, Adding chemical dimerizer: frame 5

**Figure 3:**
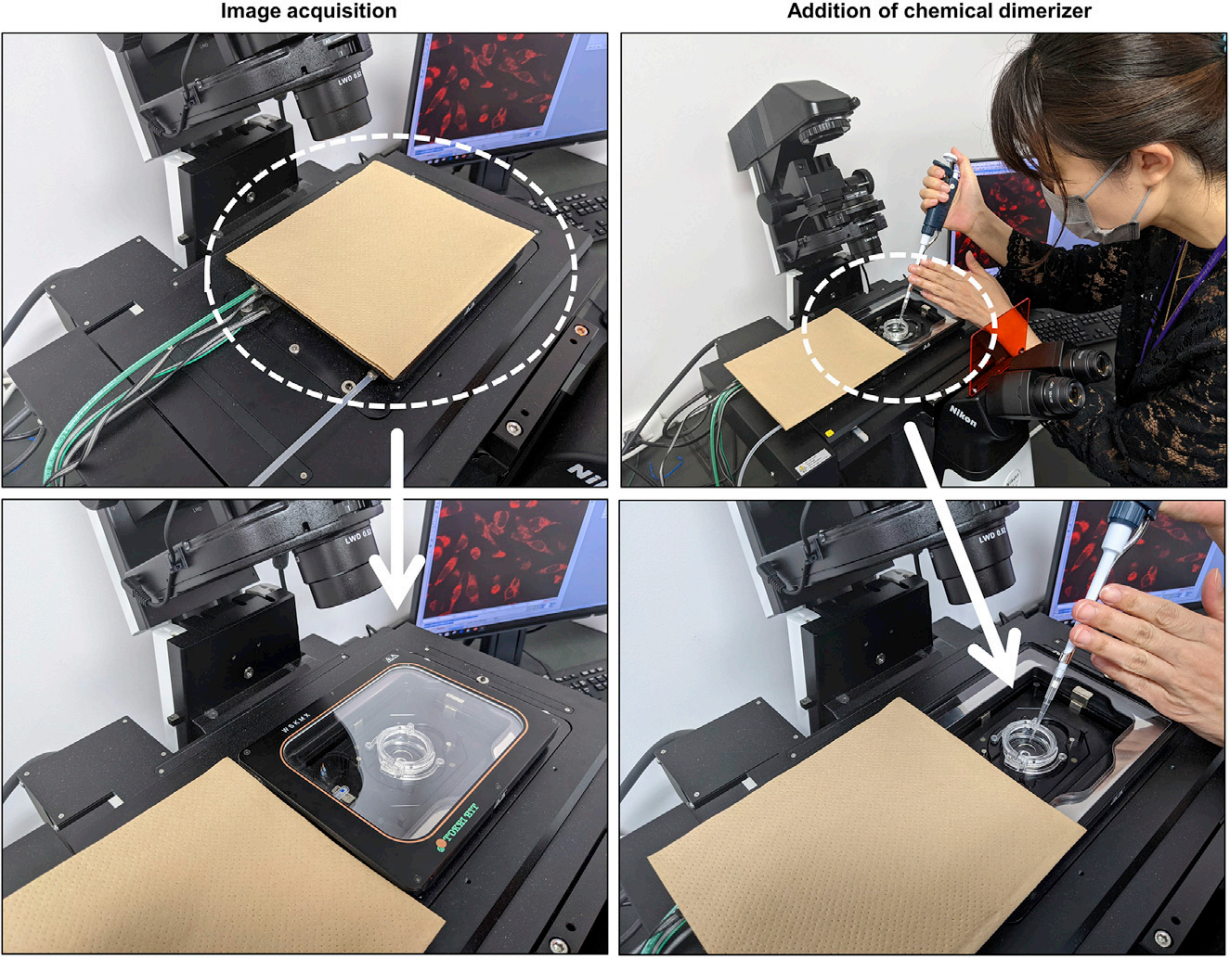
How to add chemical dimerizer during time-lapse imaging During time-lapse imaging, it is recommended that the samples are kept out of the light of the room. We create a simple dark room by shading the underside of the microscope and placing a kim towel on top of the stage-top incubator where light can enter. When adding rapamycin, the cover of the stage-top incubator is removed, and rapamycin is added. After the addition of rapamycin, the cover is closed, and the images are taken again in a simple darkroom. In this figure, the light is turned on to facilitate the photo shoot; however, in the actual experiment, the light in the room is switched off.

***Note:*** To treat the cells uniformly with rapamycin, aliquot 1 μL 100 μM rapamycin into a 1.5-mL microcentrifuge tube and suspend in 100 μl imaging medium. Add the suspension so that it is spread evenly over the entire culture dish and is not accumulated in only one place.

***Note:*** The addition of rapamycin does not necessarily have to be performed in the dark. However, it is recommended that imaging is performed in the dark.

***Note:*** It is essential to use imaging conditions that do not cause changes in mitochondrial morphology unless the iCMM is activated. To optimize imaging conditions, it is recommended to perform time-lapse imaging without adding rapamycin and confirm that mitochondrial morphology does not change (no fragmentation or aggregation of mitochondria).

26. Add a chemical dimerizer (1 μL 100 μM rapamycin in this protocol) at the appropriate time point to induce mitochondrial morphology changes (Figure 3).

27. Process the imaging data using NIS-Elements AR imaging software (Nikon).

**Figure 4:**
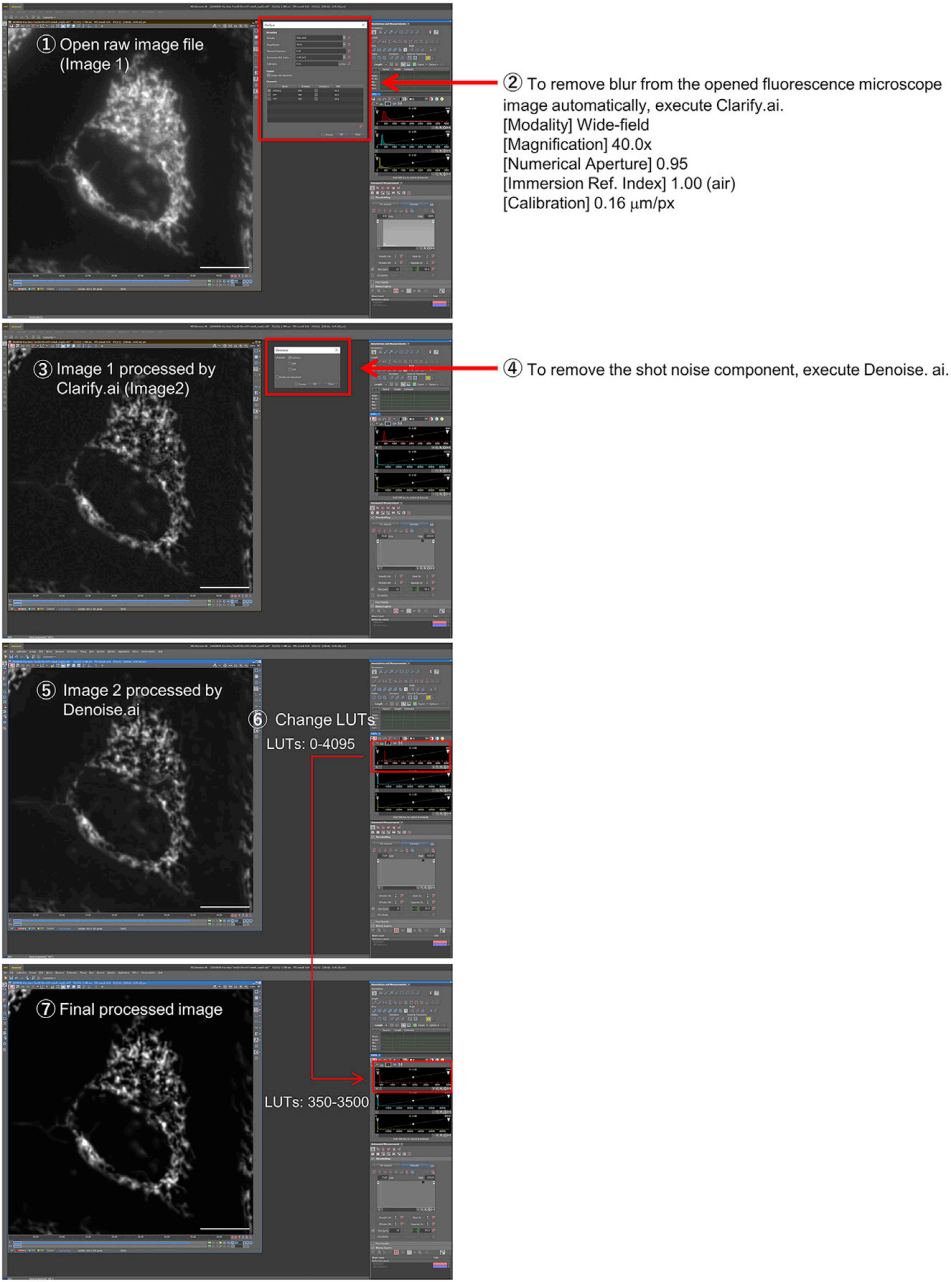
An example of image processing for mitochondria An image of the processing in NIS-Elements AR imaging software of a raw image of mitochondria in HeLa cells visualized by MitoTracker Red CMXRos dye is shown.

***Note:*** For image processing, it is necessary to follow the Data and Image Processing Policy of each journal. We show in Figure 4 the image processing to be performed for a representative mitochondrial image acquired by epifluorescence microscopy.

***Note:*** When performing quantitative analysis of mitochondrial morphology, it is necessary to perform image processing according to the method used. Methods using Image J (Valente et al., 2017) and machine learning (Leonard et al., 2015; Zahedi et al., 2018) have been developed for mitochondrial morphology analysis.

## Expected outcomes

**Figure 5:**
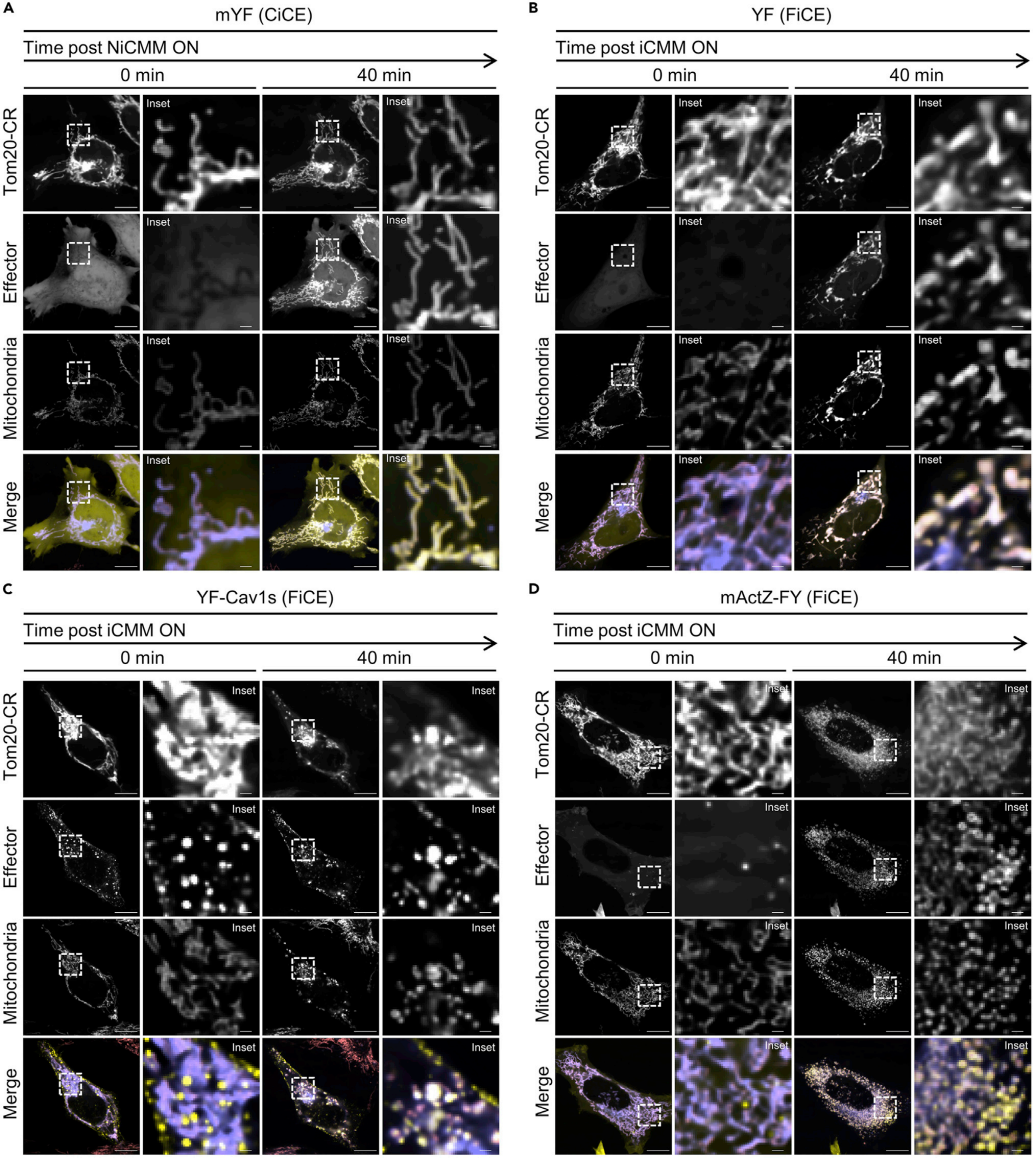
iCMM-induced changes in mitochondrial morphology Reprinted with permission from Miyamoto et al., 2021. (A–D) Time-lapse imaging (40 min/frame) of HeLa cells transiently expressing the indicated NiCMM/iCMM system was performed on confocal microscopy. Rapamycin (50 nM) was added after image acquisition at frame 1 (indicated as “0 min”). Mitochondria (coral) were stained with the MitoTracker Red CMXRos dye. Tom20-CR: slate blue, effector; yellow. Scale bar = 10 μm. Insets show a magnified view of the boxed area. Scale bar in inset = 1 μm. LUTs (Effector): 0–2000 [mYF, YF, and YF-Cav1s], 0–500 [mActZ-FY].

iCMM facilitates inducing mitochondrial morphology changes in a time scale of minutes. Mitochondrial morphology changes induced by iCMM depend on the used FiCE. Specifically, YF induces a large punctate structure of mitochondria (due to clustering rather than fusion of mitochondria), while YF-Cav1s and mActZ-FY induce a small punctate structure (Figure 5). In contrast, NiCMM, which is the technical counterpart of iCMM, does not induce any mitochondrial morphological changes, even when CiCE is translocated to the mitochondria (Figure 5). We have so far confirmed that the induction of mitochondrial morphological changes by iCMM occurs similarly in HeLa human cervix adenocarcinoma cells, Hep 3B human hepatoma cells, and U-2 OS human bone osteosarcoma cells (Miyamoto et al., 2021).

In our experiments, we confirmed that iCMM-induced mitochondrial morphology changes do not affect mitochondrial membrane potential or reactive oxygen species production over a 120-min observation window as judged by tetramethylrhodamine ethyl ester (Thermo Fisher, T669) and MitoSox (Thermo Fisher, M36008), respectively. In contrast, the oxygen consumption and extracellular acidification rates were decreased only when mitochondrial morphological changes were induced by mActZ-FY.

## Limitations

It should be noted that as iCMM artificially induces changes in mitochondrial morphology, comparisons of the respective results with physiological mitochondrial morphological changes should be made with caution. However, detailed observation of cellular responses to iCMM-induced alterations may yield novel insights. Thus, an appropriate combination of iCMM and conventional genetic approaches (e.g., modification of genes involved in mitochondrial morphology) is required to elucidate the operating mechanism(s) of living organisms, with a focus on mitochondrial morphology.

The current protocol employs rapamycin; however, it is important to note that rapamycin is a potent inhibitor of mammalian/mechanistic targeting of rapamycin complex 1, a serine/threonine kinase. If the use of rapamycin is not suitable, then other CID methods or optogenetics may be used instead (Stanton et al., 2018). However, optimization of the effectors and anchor is necessary.

## Troubleshooting

### Problem 1

No or very few cells express iCMM/NiCMM (steps 14 and 22 in step-by-step method details).

### Potential solution

With respect to cell lines that are used for the first time, it is recommended to transfect only mYF in order to optimize the transfection protocol. The amount of plasmid to be transfected (0.1–5 μg), the ratio of plasmid to transfection reagent (1:1–1:5), and the time between transfection and experiment (12–72 h) are the three most crucial points to consider. It is also possible that performing transfection 18–24 h after seeding the cells may improve the results. If there are no cells expressing iCMM/NiCMM at all, we recommend changing the transfection reagent or the transfection method (e.g., electroporation and lentivirus transfection).

By contrast, if the efficiency of gene expression is low in a cell line that has been expressing genes successfully in the past, first check the quality of the plasmid: ensure that the A260/A280 ratio is < 1.8, and that the A260/A230 ratio is between 1.8 and 2.2. In addition, run 500 ng plasmid on a 1% agarose gel by electrophoresis to ensure that the plasmid is not degraded. Next, check cell morphology and proliferation rate to determine whether cell characteristics are changed. If there are no respective issues, it is recommended to individually change the conditions and factors (Opti-MEM or transfection reagent) for stepwise examination. New reagents or media may also be tested.

### Problem 2

**Figure 6:**
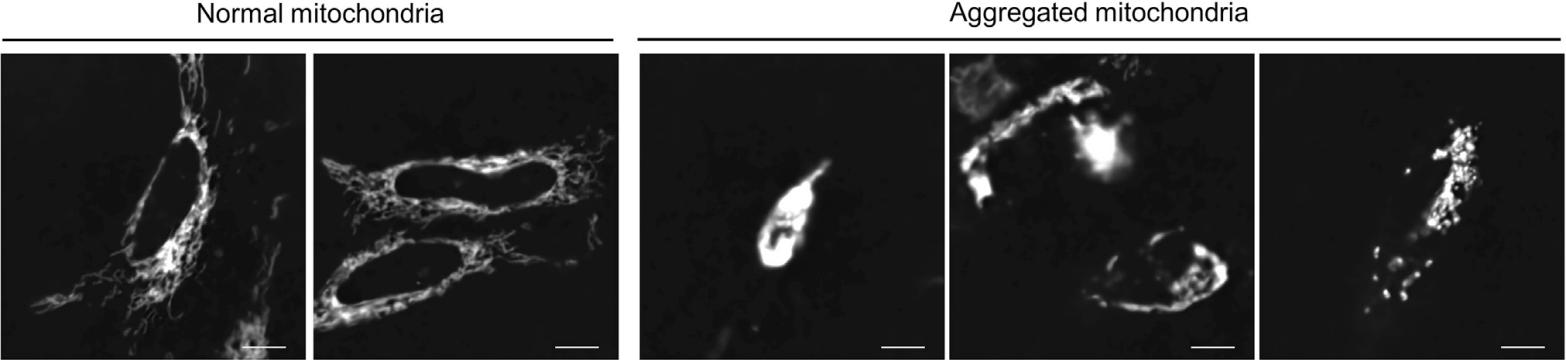
Representative images of aggregated mitochondria HeLa cells were transfected with Tom20-CR. Live-cell imaging was performed at 18 h post-transfection. Raw mitochondrial images were processed by Clarify.ai. and Denoise.ai. Scale bar = 10 μm.

The mitochondrial structure is aggregated in cells expressing iCMM/NiCMM before starting time-lapse imaging (Figure 6) (step 22 in step-by-step method details).

### Potential solution

If expression levels of Tom20-CR are excessively high, mitochondria may not be able to form a normal network structure. This can be improved by reducing the amount of plasmid introduced into the cells or by reducing the time between transfection and experiment. In addition, this may also be improved by using a mitochondria-targeting signal (MTS) other than Tom20-derived MTS or by changing the length of the linker connecting the functional domains.

### Problem 3

The number of cells adhering to the glass bottom dish is low (step 22 in step-by-step method details).

### Potential solution

It is possible that cells detach during washing. Although the proteins constituting iCMM/NiCMM do not induce cell death, transfection-induced cell death occurs at a certain rate. Optimization of cell confluence is also recommended as a potential solution.

### Problem 4

The mitochondrial structure does not change after adding chemical dimerizer (step 26 in step-by-step method details).

### Potential solution

Ensure that expression levels of FiCEs and of the anchor (Tom20-CR) are adequate. For example, YF may not induce mitochondrial morphological changes effectively at low expression levels. mActZ-FY has lower expression levels than other FiCEs, however, lower expression levels are more effective for inducing mitochondrial morphology changes.

If expression of FiCE and of the anchor is sufficient, add additional chemical dimerizers. If an additional chemical dimerizer induces mitochondrial morphological changes, it is possible that the previous chemical dimerizer was not added adequately. Further, ensure that the chemical dimerizer had been stored properly.

### Problem 5

Fluorescence intensity of mitochondria stained with MitoTracker Red CMXRos decrease during time-lapse imaging (step 27 in step-by-step method details).

### Potential solution

Change the exposure time and ND filter to achieve the lowest fluorescence intensity at which mitochondria can be observed or reduce the number of imaging positions. As MitoTracker Red CMXRos stains mitochondria in a mitochondrial membrane potential-dependent manner, it is also necessary to avoid imaging under conditions in which the membrane potential is reduced.

## Resource availability

### Lead contact

Further information and requests for resources and reagents should be directed to and will be fulfilled by the lead contact, Takafumi Miyamoto (takmi565@md.tsukuba.ac.jp).

### Materials availability

Plasmids generated in this study have been deposited to Addgene (see key resources table).

## Data Availability

This study did not generate any datasets or code.
